# Genome empowerment for the Puerto Rican parrot – *Amazona vittata*

**DOI:** 10.1186/2047-217X-1-13

**Published:** 2012-09-28

**Authors:** Stephen J O’Brien

**Affiliations:** 1 Theodosius Dobzhansky Center for Genome Bioinformatics, St. Petersburg State University, St. Petersburg, Russia

**Keywords:** Puerto Rican parrot, Whole-genome sequencing, Genomics, Conservation, Education, Funding

## Abstract

A unique community-funded project in Puerto Rico has launched whole-genome sequencing of the critically endangered Puerto Rican Parrot (*Amazona vittata*), with interpretation by genome bioinformaticians and students, and deposition into public online databases. This is the first article that focuses on the whole genome of a parrot species, one endemic to the USA and recently threatened with extinction. It provides invaluable conservation tools and a vivid example of hopeful prospects for future genome assessment of so many new species. It also demonstrates inventive ways for smaller institutions to contribute to a field largely considered the domain of large sequencing centers.

## Main text

Perhaps one of the more gratifying aspects of the post-genomics era is marveling at the creativity of individual projects that push the envelope further and further over the edge. Witness the emergence of human “copy number variation” and discerning that their segmental aneuploidy might affect gene dosage and explain a few hereditary diseases (it does). Or 23andMe, the upstart SNP genotyping-for-the-people venture that began by predicting Oprah Winfrey’s curious ancestry and now is immersed in personal medical genomics disclosure for an affordable price. Or this month’s ENCODE bombshell that features some 4-million new gene regulatory sequence stretches amidst the sea of noncoding genomic DNA (98% of human DNA formerly dubbed “junk DNA”; well hardly!) [1].

An article published alongside this paper in *GigaScience* this month unfolds yet another novel genomics-stimulated innovation — a unique grassroots endeavor to sequence the genome of a critically endangered species from a remote locale where the species survives and is empowered by the local citizenry who wanted to help [2]. The Puerto Rican parrot’s genome has been sequenced and assembled; annotation has commenced and the fresh new data (29-fold Illumina coverage) sits in an open access *GigaScience* database, *Giga*DB [3] and a genome browser for any party to query and improve. The work was led by Taras Oleksyk, Juan Carlo Martiez-Cruzado, along with a coterie of conservation minded scientists, and their students at the University of Puerto Rico-Mayaguez – not really a hotbed of genome sequencing centers.

**Figure 1:**
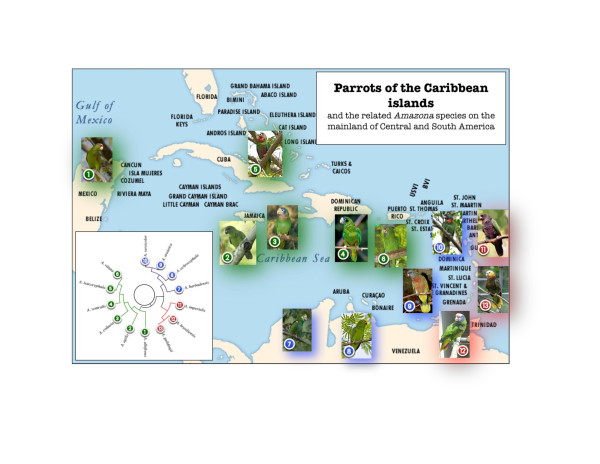
**Geographic dispersal of parrot species of the genus *Amazona *on Greater and Lesser Antilles.** Amazon parrots in the Caribbean are classified into three taxonomic groups. **(Green)** The Greater Antillean group related to (1) white-fronted amazon (*Amazona albifrons*) of Central America, includes (2) black-billed (*A. agilis*), (3) yellow-billed (*A. collaria*), (4) Cuban (*A. leucocephala*), Hispaniolan amazon (*A. ventralis*) and (6) Puerto Rican parrot (*A. vittata*). The Lesser Antillean Amazons are derived from two different colonization events, both from South America. The first group **(Blue)** is related to the (7) yellow-shouldered (*A. barbadensis*), and the (8) yellow-crowned (*A. ochrocephala*) amazons, and includes (9) red-necked amazon (*A. arausiaca*), and (10) St. Lucia’s parrot (*A. versicolor*). The second group **(Red)**, is related to the (12) red-tailed amazon (*A. brasiliensis*), and includes (11) the imperial (*A. imperialis*), and (13) St. Vincent amazon (*A. guilingii*). **Insert:** A phylogram showing the relationship between the eight extant *Amazona* species of the Caribbean and their closest relatives on the mainland. Illustration courtesy of Taras K Oleksyk.

**Figure 2:**
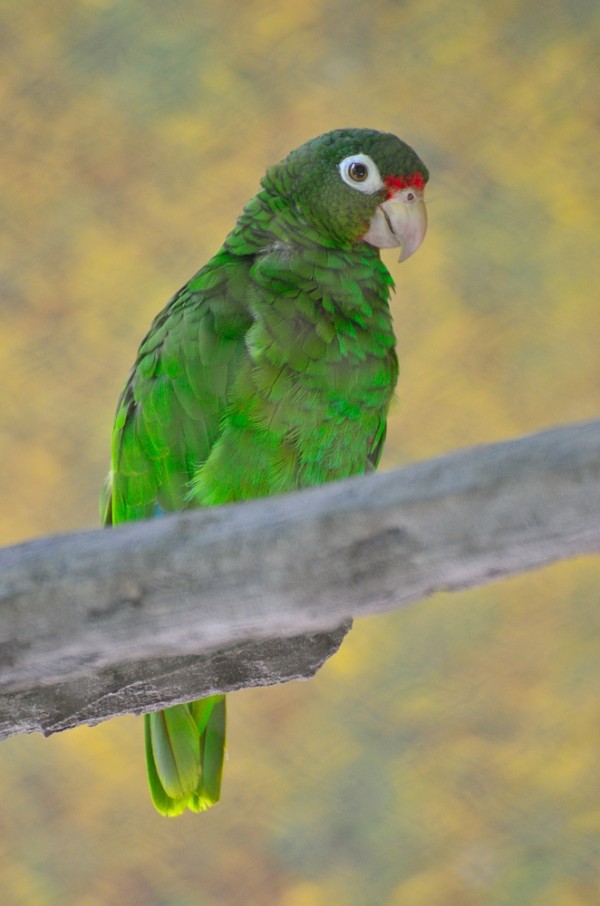
**Puerto Rican parrot at the new Puerto Rican Zoo Juan A Rivero exhibit in Mayaguez.** Photograph by Jose Almodovar.

The Puerto Rican parrot is a uniquely American parrot, but one of eight parrot species found in the Caribbean (30 species comprise the 4 million years (MY) old-genus radiation in the Amazon region), a graphic example of speciation via island biogeography (Figure 1 and Figure 2). The species was listed as endangered by the US Fish and Wildlife Service in 1967 and as critically endangered by the International Union for Conservation of Nature (IUCN) Red Book in 1994. Endemic and restricted to Puerto Rico, the Puerto Rican parrot wild population was approaching extinction in 1972 when the population fell to 16 birds. A captive breeding program was established and guided by the legendary Ulysses S. Seal through a 1988 Population Viability Analysis sponsored by the Conservation Breeding Specialist Group of the IUCN [4]. A steady and deliberate increase in fertile mate pairs, fertile eggs, and fledglings resulted in over 100 parrots released to or born in the wild by 2011. 

The monitoring of these recovery releases in the wild populations as well as managing captive breeding programs will benefit considerably from the tools derived from this new genome sequence and annotation, particularly SNP, indel, and microsatellite variants that resolve kinship, historic migration, inbreeding, parentage, and dynamic population structure.

The genome of the Puerto Rican parrot was estimated at ~1.58 Gbp, about half the size of the human genome. The light 29x coverage reflected some 76% of the genome, and contigs assembled with Ray and SOAPdenovo were joined into scaffolds of modest size (N50 ~19.5 kb) using two insert libraries (300 bp and 2.5 kb inserts). The authors aligned their scaffolds to the more advanced genome assemblies of chicken and zebra finch (which are a long way away in evolutionary time, *circa* 90MY, as far as humankind has diverged from a common ancestor with mice). They annotated repeat families, but do not yet present a framework map to discriminate gene organization (vice zebra finch or chicken), a gene assessment, a description of SNP variation, a listing of microsatellite loci, Numts, microRNAs, endogenous retroviruses, nor other features that users of genome sequence thirst for. This work is a raw and preliminary effort, but a welcome starting gun for genomics and conservation communities to rapidly supply the finer genome feature details.

The achievement of the assembled Puerto Rican parrot genome is an important milestone for unusual reasons. First, the genome project was funded by student organized art and fashion shows dedicated to the effort plus scores of small personal donations by Puerto Rican people who wanted to be part of it. That could only happen when the cost of reagents had dropped so precipitously that it can be afforded within a $10,000 USD budget. Second, the analysis and annotation took place in a modest university setting where students of genome bioinformatics were trained to drive assemblers, to stitch together contigs and scaffolds, and to begin the genome annotation process. Third, the Puerto Rican parrot is a harbinger for the many parrot genomes we shall be seeing in the near future: the opportunity to explore speciation and adaptive radiation among island species of these parrots is too tempting to pass up (Figure 1). The Genome 10 K sponsored Assemblathon-II (led by Ian Korf) ( http://assemblathon.org) is evaluating assembly strategies for three vertebrate species: a cyclid fish, a python, and a parakeet songbird (*Melopsittacus undulatus*) [5]. And great things are expected from the BGI-Genome 10 K Avian phylogenomics consortium (led by Erich Jarvis) annotating the genomes of 48 plus avian species including more parrots, such as the Kea (*Nestor notabilis*) coming later in 2012 [6].

It may be a telling coincidence that only a few weeks have passed since the genome sequence and assembly of *Geospiza fortis*: the iconic species better known as Darwin’s finch was accomplished (by the BGI) [7], announced and released on the UC Santa Cruz Genome Browser ( http://genome.ucsc.edu) prior to publication of the primary analysis paper to promote rapid data use. Evolutionary genomics has gotten a boost this month to be sure.

## Conclusions

The genomics community has expanded of late from anthropocentric emphasis to enormous enthusiasm for the comparative genome sequence achievement of thousands of species. The Genome10K Project [8] is poised to assist and facilitate the genome sequencing, assembly, and annotation of 10,000 vertebrate species in the near future. Ditto for the Insect 5 K project for 5,000 insect species, and the fledgling Global Invertebrate Genomics Alliance (GIGA) for invertebrate species [9]. If these projects are successful, we will need genome-science bioinformaticians for 25,000 species rather soon. Will they be supplied by the traditional genome sequencing centers, by mega-sequencing centers as for the BGI, or by young scientists across the globe like those trained on the Puerto Rican parrot’s genome, poised to make sense, aka a comprehensive genome browser for each new species’ sequence? Time will tell, but I have my suspicions.

## Abbreviations

bp: Base pair; IUCN: International Union for Conservation of Nature; MY: Million years.

## Competing interests

The author declares he has no competing interests.

## Authors’ information

The author is Co-Director of the Genome 10K Project and Chief Scientific Officer of Theodosius Dobzhansky Center for Genome Bioinformatics, St. Petersburg State University, Russia. He knows more about cat genomics than about bird genomics.
