# Correction: Genome empowerment for the Puerto Rican parrot – *Amazona vittata*

**DOI:** 10.1186/2047-217X-1-17

**Published:** 2012-11-27

**Authors:** Stephen J O’Brien

**Affiliations:** 1 Theodosius Dobzhansky Center for Genome Bioinformatics, St. Petersburg State University, St. Petersburg, Russia

## Correction

In the article ‘Genome empowerment for the Puerto Rican parrot – *Amazona vittata. GigaScience* 2012 1:13’ [1] the leadership of avian phylogenomic project [2] mentioned in commentary should have been Guojie Zhang, Erich Jarvis, and Tom Gilbert [2]. We regret this omission.
